# Glutamine deprivation stimulates mTOR-JNK-dependent chemokine secretion

**DOI:** 10.1038/ncomms5900

**Published:** 2014-09-25

**Authors:** Naval P. Shanware, Kevin Bray, Christina H. Eng, Fang Wang, Maximillian Follettie, Jeremy Myers, Valeria R. Fantin, Robert T. Abraham

**Affiliations:** 1 Oncology Research Unit, Pfizer Worldwide Research and Development, 401 N. Middletown Road, Pearl River, New York 10965, USA; 2 Oncology Research Unit, Pfizer Worldwide Research and Development, 10777 Science Center Drive, La Jolla, California 92121, USA

## Abstract

The non-essential amino acid, glutamine, exerts pleiotropic effects on cell metabolism, signalling and stress resistance. Here we demonstrate that short-term glutamine restriction triggers an endoplasmic reticulum (ER) stress response that leads to production of the pro-inflammatory chemokine, interleukin-8 (IL-8). Glutamine deprivation-induced ER stress triggers colocalization of autophagosomes, lysosomes and the Golgi into a subcellular structure whose integrity is essential for IL-8 secretion. The stimulatory effect of glutamine restriction on IL-8 production is attributable to depletion of tricarboxylic acid cycle intermediates. The protein kinase, mTOR, is also colocalized with the lysosomal membrane clusters induced by glutamine deprivation, and inhibition of mTORC1 activity abolishes both endomembrane reorganization and IL-8 secretion. Activated mTORC1 elicits *IL8* gene expression via the activation of an IRE1-JNK signalling cascade. Treatment of cells with a glutaminase inhibitor phenocopies glutamine restriction, suggesting that these results will be relevant to the clinical development of glutamine metabolism inhibitors as anticancer agents.

## Abstract (web summary)

Glutamine deprivation is currently being tested as a therapeutic strategy in cancer. Shanware *et al.* show that in cultured cells, glutamine deprivation stimulates IL-8 secretion by triggering endoplasmic reticulum stress, and suggest that the potential of this effect to influence tumour development should be examined.

## Graphical abstract

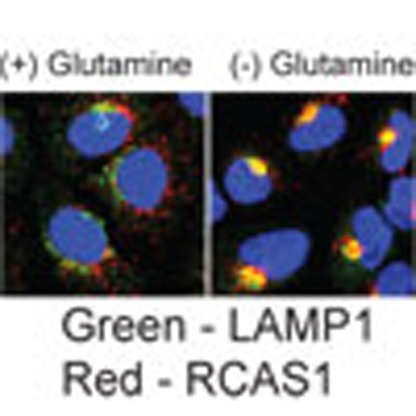

Reprogramming of molecular and metabolic pathways involved in intermediate metabolism is now recognized as a hallmark of cancer^1^. Oncogenic signals drive constitutive cell growth and proliferation, and place heavy demands on the pathways responsible for providing the metabolic building blocks needed for the synthesis of proteins, nucleic acids, lipids and other macromolecules. To meet the increased demand for biosynthetic precursors, cancer cells increase uptake of glucose and other nutrients, and shift overall metabolism from bioenergy (ATP) production and cell maintenance activities to anabolic processes that support cell mass accumulation and mitotic cell division^2^,^3^.

The shift toward anabolic metabolism is exemplified by the altered catabolism of glucose in tumour tissues^4^. Normal, non-proliferating cells primarily convert glucose to pyruvate via glycolysis. Pyruvate is then imported into the mitochondria, where it is converted into acetyl CoA for entry into the tricarboxylic acid (TCA) cycle. The glucose-derived carbon is then completely oxidized to produce carbon dioxide and ATP. In contrast, tumour cells reduce pyruvate to lactate for export from the cells. The glycolytic breakdown of glucose to lactate in oxygenated tumour tissues is termed the Warburg effect^5^. In addition to lactate, glycolysis generates intermediates that fuel anabolic metabolism via the pentose-phosphate and serine biosynthesis pathways^4^. Similarly, the TCA cycle is involved in both energy production and in the generation of building blocks for protein and lipid biosynthesis. The diversion of glucose-derived carbon away from the mitochondria, together with the withdrawal of TCA cycle intermediates for biosynthetic reactions, creates a carbon deficit in the TCA cycle that must be corrected by entry of carbon from other sources, a process termed anaplerosis^6^. These and other alterations in nutrient uptake and utilization in transformed cells have spawned considerable interest in cancer metabolism as a promising area for the discovery of novel antitumour agents^7^,^8^.

The non-essential amino acid, glutamine, is a major contributor to anaplerotic replenishment of the TCA cycle, and serves as a source of carbon and nitrogen for the synthesis of proteins, lipids and amino acids^9^,^10^. Proliferating cells avidly import extracellular glutamine, and catabolize it via glutaminolysis, during which glutamine undergoes sequential deamination in the mitochondria to glutamate and further into the TCA cycle intermediate, α-ketoglutarate (α-KG)^11^. As a nitrogen donor, glutamine supports both nucleotide and non-essential amino acid synthesis, in addition to protein glycosylation through the hexosamine pathway^12^. Finally, glutamine plays a key role in oxidative stress resistance by serving as a source of glutamate for the production of glutathione^9^. Many cancer cells exhibit strikingly increased rates of glutamine uptake and metabolism. Notably, cells transformed by the *MYC* proto-oncogene or oncogenic *KRAS* display glutamine auxotrophy^13^,^14^,^15^. The increased sensitivity of certain transformed cells to glutamine restriction suggests that drugs interfering with glutamine catabolism might have clinically exploitable antitumour activities^16^,^17^. An actionable target for such inhibitors is the mitochondrial enzyme, glutaminase, which catalyses the conversion of glutamine to glutamate. Clearly, our understanding of the potential benefits and challenges of therapeutic targeting of glutamine metabolism in cancer patients will benefit from a more complete understanding of the cellular responses to manipulations that deprive cancer cells of glutamine or interfere with glutaminolysis.

We and others have recently described an unanticipated contribution of glutaminolysis to autophagy, a cytoplasmic pathway that delivers autophagosome-encapsulated macromolecules and organelles to lysosomes for degradation and recycling into metabolic processes^18^,^19^,^20^,^21^. Cells normally exhibit a basal level of autophagic flux that is strongly enhanced by certain environmental stresses, such as nutrient starvation. Under such stressful conditions, autophagy allows cells to degrade non-essential macromolecules into products that support cellular bioenergetics and viability^22^. A recent manuscript by Narita *et al.*^23^ also identified an essential role for autophagy in the context of a multi-component endomembrane structure termed the TOR-autophagy spatial coupling complex (TASCC). In cells undergoing oncogene-induced senescence (OIS), the TASCC is formed by the spatial colocalization of the autophagy machinery with lysosomes, and appears to facilitate the mass synthesis of secretory proteins that comprise the senescence-associated secretory phenotype (SASP).

In this report, we demonstrate that short-term glutamine restriction results in a chemokine-secretory response that is dependent on the induction of the ER stress-response pathway. Glutamine restriction-induced ER stress triggers the reorganization of subcellular organelles into a spatially localized, cytoplasmic compartment that supports a robust autophagic response, together with activation of the mechanistic target of rapamycin complex 1 (mTORC1). These events, in turn, drive the expression and secretion of the pro-inflammatory cytokine, IL-8. Treatment of the cells with a glutaminase inhibitor phenocopies these responses, suggesting that the acquisition of a secretory phenotype may have implications for therapeutic strategies aimed at interfering with tumour-associated glutamine metabolism in cancer patients.

## Results

### Glutamine deprivation induces the ER stress response

In initial studies, we profiled the transcriptional changes induced by short-term (24 h) glutamine deprivation in U2OS osteosarcoma cells. Cell growth was partially reduced, with no appreciable differences in cell viability induced by this glutamine restriction protocol (Supplementary Fig. 1). Microarray analysis of gene expression in glutamine-restricted U2OS cells revealed transcriptional changes associated with increased endoplasmic reticulum (ER) stress, lysosomal activity, autophagy and increased expression of several cytokines that have been linked to the SASP (Table 1).

**Figure 1:**
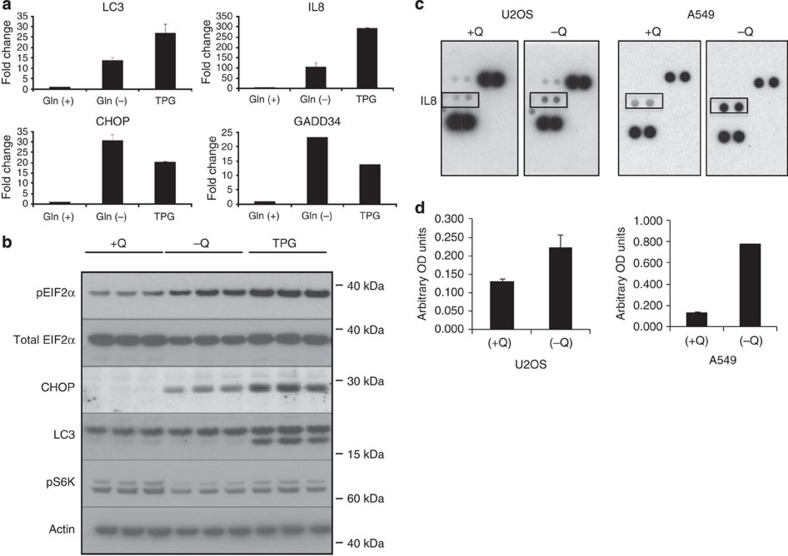
Glutamine deprivation induces IL-8 secretion. (**a**) RT–PCR analysis of LC3, IL-8, CHOP and GADD34 in U2OS cells subjected to glutamine deprivation (−Q) or exposed to TPG (1 μM). (**b**) Immunoblot analysis of ER stress- and autophagy-related proteins in U2OS cells subjected to glutamine deprivation or TPG treatment. Samples are run in triplicate. (**c**) Cytokine array analysis of conditioned media from U2OS and A549 cells grown in the presence (+Q) or absence (−Q) of glutamine. (**d**) IL-8 enzyme-linked immunosorbent assay of conditioned media from U2OS and A549 cells grown in the presence (+Q) or absence (−Q) of glutamine. Error bars in all figures represent s.d. of three biological replicates.

The ER stress-response pathway is triggered by the accumulation of unfolded or misfolded proteins in the ER lumen, leading to attenuation of global protein translation, accompanied by selective increases in the transcription and translation of several chaperone and stress-related proteins^24^,^25^. We observed robust induction of canonical ER stress-responsive genes *DDIT3* (also known as CHOP or GADD153) and *PP1R15A* (GADD34) during glutamine deprivation (Table 1). To confirm these results, we performed quantitative reverse transcription PCR (RT–PCR) analyses of mRNA derived from U2OS cells that were either glutamine deprived or treated with thapsigargin (TPG), an established ER stress inducer (Fig. 1a). Increased CHOP and GADD34 levels were observed in both glutamine-deprived and TPG-treated cells. Thus, glutamine restriction stimulated a gene expression programme that overlapped with that triggered by the canonical ER stress inducer, TPG. Immunoblot analyses revealed that glutamine-deprived cells exhibited increased eukaryotic translation initiation factor 2α (eIF2α) phosphorylation and CHOP protein expression, two hallmarks of the ER stress response^25^ (Fig. 1b).

Microarray analysis also revealed elevated expression of several autophagy-related genes, including LC3 (MAP1LC3B), WIPI1 and UVRAG, and increased expression of several subunits of the vacuolar ATPase (v-ATPase), a protein complex required for lysosomal acidification, lysosome-dependent protein degradation and mTORC1 activation (Table 1)^26^. Notably, we found increased expression of subunit V_1_C_1_ (ATP6V1C1), a rate-limiting component of the v-ATPase enzyme complex^27^. RT–PCR confirmed the transcriptional induction of the autophagy-related gene LC3 suggesting that glutamine restriction upregulated autophagy in these cells (Fig. 1a).

Unexpectedly, glutamine deprivation stimulated the expression of genes encoding cytokines previously associated with the SASP response (Table 1)^28^. In repeat experiments in multiple cell lines, we observed that the increased interleukin-8 (IL-8; also known as CXCL8) mRNA was the most consistent SASP-related response to glutamine deprivation. Therefore, we focused our subsequent characterization efforts on *IL8* (Table 1). Glutamine restriction-induced *IL8* transcription was confirmed by RT–PCR (Fig. 1a). To profile the effects of glutamine restriction on cytokine secretion, we analysed conditioned medium from glutamine-deprived U2OS cells, as well as A549 lung cancer cells. Increased levels of IL-8 secretion were detected in the conditioned medium from both cell lines (Fig. 1c,d).

### Glutamine deprivation induces autophagy

**Figure 2:**
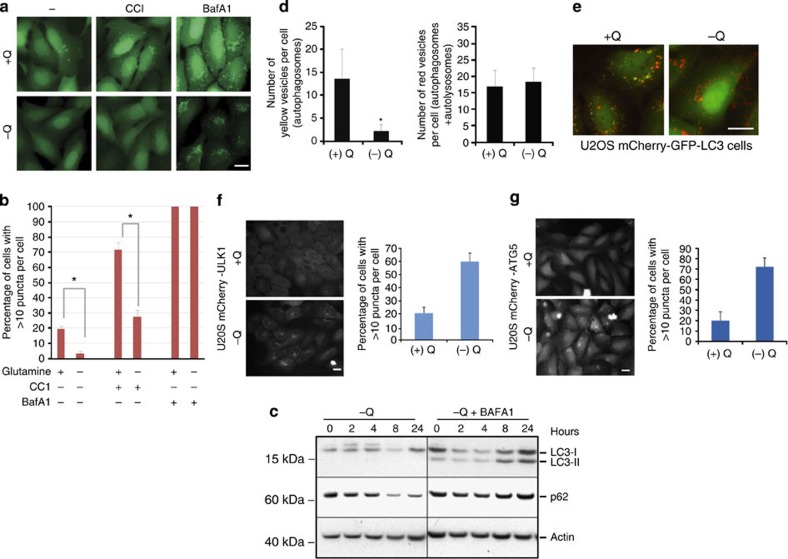
Glutamine deprivation induces autophagic flux. (**a**) Autophagosomes were visualized in U2OS cells stably expressing a GFP-LC3 reporter construct. GFP-labelled puncta were examined after 24 h in the presence (+Q) or absence (−Q) of glutamine, with or without co-addition of 100 nM CCI-779 (CCI) and 400 nM BafA1. Scale bar, 10 μm. (**b**) Graphical summary of experiments performed as described in **a**. Percentage of cells with >10 puncta per cell from three independent experiments is depicted. Bars represent mean±s.d. from three independent experiments (>50 cells per experiment). The statistical significance (*P* value) was determined by a two-tailed, paired Student’s *t*-test. *P<0.05. (**c**) Immunoblot analysis of U2OS cells subjected to glutamine deprivation with or without 400 nM BafA1. Cells were pretreated with BafA1 for 1 h before and during exposure to glutamine-deficient medium. Autophagic activity was monitored by detection of p62 and LC3-II proteins. (**d**) U2OS mCherry-GFP-LC3 cells were cultured in the presence (+Q) or absence (−Q) of glutamine for 18 h. Red vesicles denote autolysosomes, whereas yellow vesicles represent autophagosomes. Bars indicate numbers of yellow vesicles (autophagosomes) or red vesicles (autolysosomes) per cell±s.d. (**e**) Images of U2OS mCherry-GFP-LC3 cells cultured for 18 h in the presence (+Q) or absence (−Q) of glutamine. Scale bar, 10 μm. (**f**) Phagophore formation in mCherry-ULK1. Scale bar, 10 μm. (**g**) mCherry-ATG5-expressing U2OS cells after 24 h in the presence (+Q) or absence (−Q) of glutamine. Scale bar, 10 μm.

Microarray analysis revealed increased expression of lysosomal and autophagy-related genes following 24 h glutamine withdrawal (Table 1). To test whether these transcriptional changes were consistent with the induction of an autophagic response, autophagosomes were visualized by the appearance of green fluorescent protein (GFP)-labelled autophagosomes in U2OS cells stably expressing GFP-tagged LC3. Cells treated with complete, serum-containing medium lacking only glutamine exhibited a clear reduction in the number of GFP-LC3-positive puncta at 8 and 24 h (Fig. 2a,b; Supplementary Fig. 2A,B). Similarly, lower numbers of fluorescent autophagosomes were observed in glutamine-deprived cells treated with the mTORC1 inhibitor, CCI-779 (ref. 29)^29^ (Fig. 2a,b). A reduction in the number of steady-state autophagosomes during glutamine deprivation could reflect either a reduced rate of autophagosome formation, or an accelerated rate of autophagosome turnover due to an increase in the number of fusion events with lysosomes. To distinguish between these possibilities, cells were subjected to glutamine deprivation in the presence of bafilomycin A1 (BafA1), a vacuolar H+ ATPase (v-ATPase) inhibitor that indirectly interferes with autophagosome–lysosome fusion and blocks autophagosome turnover^30^. BafA1 provoked a dramatic increase in GFP-LC3-positive puncta in glutamine-deprived cells, consistent with increased autophagic flux (Fig. 2a,b).

To examine whether increased autophagosome turnover was accompanied by a concomitant increase in the degradation of autophagic cargo, we monitored the levels of a well-established autophagy substrate protein, p62 (refs 30, 31). Glutamine starvation resulted in a substantial, time-dependent reduction in p62 levels (Fig. 2c), in spite of the increase in p62-encoding mRNA transcripts under these conditions (Table 1). The reduction in p62 levels in the glutamine-deprived cells was reversed in the presence of BafA1, indicating that p62 reduction was attributable to increased autophagic flux (Fig. 2c). In addition, BafA1 treatment was required to visualize the mature, lipidated LC3-II band in glutamine-deprived cells, indicating that glutamine restriction increased the turnover of LC3-II in autolysosomes (Fig. 2c). This conclusion was further substantiated by studies comparing the effects of glutamine restriction on p62 in autophagy-competent *Atg5*+/+ immortalized baby mouse kidney (iBMK) cells with that seen in autophagy-defective *Atg5*−/− iBMK cells^32^,^33^. Glutamine withdrawal resulted in a reduction in p62 in *Atg5*+/+ cells, but not in their *Atg5−*/− counterparts (Supplementary Fig. 2C). Importantly, BafA1 reversed the reduction in p62 only in the autophagy-competent *Atg5*+/+ cells. Finally, we used the mCherry-GFP-LC3 dual-reporter assay^30^ that allows discrimination of autophagosomes from autolysosomes. In this assay, autophagosomes are visualized as GFP- and mCherry-positive (yellow) puncta, whereas autolysosomes exhibit only the mCherry (red) signal due to quenching of GFP fluorescence in the acidic lysosomal compartment. Glutamine deprivation led to a decrease in the number of yellow vesicles (autophagosomes) with no appreciable reduction in the number of red vesicles (autophagosomes+autolysosomes), consistent with enhanced autophagosome to autolysosome turnover (Fig. 2d,e). We also tested the effect of glutamine deprivation on phagophore formation. ULK1 and ATG5 are involved in the early steps of autophagy, and are physically associated with the phagophore but not retained on the mature autophagosome^34^. The expression of fluorochrome-tagged versions of these two proteins in cells therefore allows monitoring of microscopically visible phagophores and immature autophagosomes. Indeed, glutamine withdrawal in U2OS cells expressing mCherry-ULK1 or mCherry-ATG5 fusion proteins stimulated the appearance of mCherry-positive puncta (Fig. 2f,g). In contrast, exposure to BafA1 did not stimulate the appearance of these puncta, even though it led to autophagosome accumulation (Supplementary Fig. 2D,E). The increases in ULK1- and ATG5-positive puncta indicated that glutamine deprivation induced autophagy early in the autophagic process. Collectively, these data provide strong evidence that glutamine deprivation stimulated autophagosome formation, turnover and cargo degradation.

### Colocalization of the autophagy and secretory machinery

**Figure 3:**
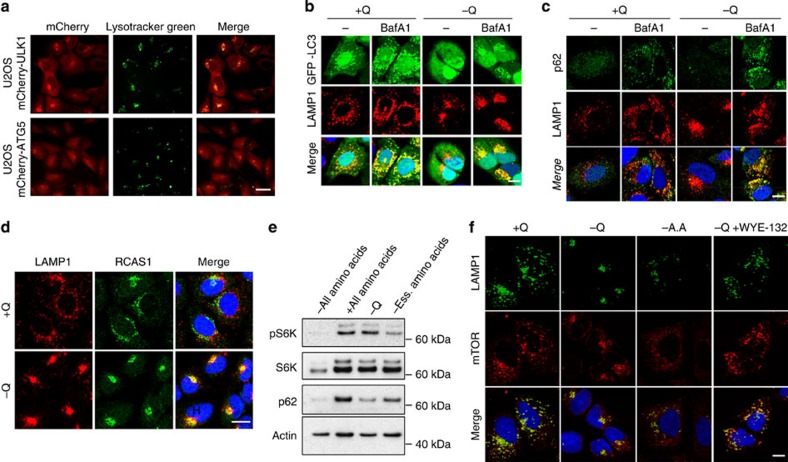
Glutamine deprivation induces endomembrane clustering and colocalization of mTORC1. (**a**) Colocalization of lysosomes (Lysotracker Green) with phagophores (mCherry-ULK1 and mCherry-ATG5 puncta) in glutamine-deprived U2OS cells. Scale bar, 25 μm. (**b**) Colocalization of LAMP1-positive lysosomes with GFP-LC3-labelled autophagosomes after glutamine deprivation in the absence or presence of 400 nM BafA1 in U2OS cells. Scale bar, 15 μm. (**c**) Immunofluorescence images of U2OS cells stained for p62 expression and LAMP1 following glutamine deprivation and BafA1 treatment. Nuclei were stained with DAPI. Scale bar, 15 μm. (**d**) Immunofluorescence images of cells stained with LAMP1 (lysosomes) and the Golgi marker, RCAS1, following glutamine deprivation. Nuclei were stained with DAPI. Scale bar, 15 μm. (**e**) U2OS cells were treated for 24 h with cell culture medium containing the indicated amino acids. Cell lysates were immunoblotted to detect changes in S6K phosphorylation and p62. (**f**) Immunofluorescence staining for LAMP1 (green) and mTOR (red) in U2OS cells subjected to glutamine deprivation (24 h), in the presence of 100 nM WYE-125132 (WYE-132). −AA denotes cells deprived of all amino acids. Scale bar, 15 μm. Error bars in all figures represent s.d. of three biological replicates. Ess., essential.

In light of the endomembrane reorganization, and the accompanying increases in autophagy and IL-8 secretion induced by glutamine restriction, we hypothesized that these endomembrane clusters might be similar to the TASCC structure described by Narita *et al.*^23^ We first determined whether glutamine deprivation resulted in a change in lysosomal localization. Indeed, U2OS cells showed a dramatic shift in the lysosomal staining pattern after glutamine restriction, from diffuse to a focal, perinuclear clustering (Supplementary Fig. 3A). We next examined whether these lysosomal clusters overlapped with the autophagosomal clusters noted in Fig. 2. mCherry-ULK1- and ATG5-positive puncta were extensively colocalized with lysosomes in both cell lines following glutamine restriction (Fig. 3a). Colocalization of ATG5- and LAMP1-positive endomembranes was also seen in H4 (neuroblastoma) and HCT116 cells (Supplementary Fig. 3B).

To determine whether the endomembrane reorganization response was unique to glutamine restriction, we examined lysosome distribution after withdrawal of the essential amino acid, leucine. Lysosomal clustering was evident in the leucine-starved cells, but considerably less universal on a per cell basis than removal of glutamine (Supplementary Fig. 3C,D). Leucine deprivation was correspondingly less effective than glutamine restriction with regard to autophagy induction, as measured by its effects on p62 expression (Supplementary Fig. 3E). In complete contrast to glutamine withdrawal, leucine deprivation failed to induce IL-8 gene expression, despite robust induction of the ER stress marker, CHOP (Supplementary Fig. 3F). These results suggest that clustering of the autophagic machinery with lysosomes was causally related to the increase in IL-8 production observed in glutamine-restricted cells.

We had previously observed fewer GFP-LC3-positive autophagosomes in glutamine-deprived U2OS cells (see Fig. 2). We speculated that the spatial approximation of autophagic vesicles with lysosomes accelerated autophagic trafficking to lysosomes, a scenario that could explain the reduction in mature autophagosomes observed after glutamine restriction. To explore this possibility, GFP-LC3-expressing U2OS cells were subjected to glutamine deprivation in the presence of BafA1 to visualize autophagosomes that accumulated due to impaired lysosomal fusion and degradative activity. BafA1 treatment caused the appearance of GFP-LC3-positive puncta that were colocalized with LAMP1-positive lysosomal clusters in glutamine-deprived cells (Fig. 3b). BafA1 treatment also increased the colocalization of p62 with lysosomes in these cells (Fig. 3c). These findings support the hypothesis that glutamine deprivation stimulates autophagic flux, in part through colocalization of the autophagy induction and maturation apparatus with lysosomes.

The induction of SASP-related genes together with the formation of a TASCC-like subcellular structure^23^ prompted us to examine the potential involvement of the Golgi apparatus in response to glutamine restriction. The Golgi apparatus plays key roles in the sorting of newly translated proteins into various intracellular compartments and the secretory pathway, and thus may play an active role in glutamine deprivation-induced IL-8 secretion. Using RCAS1 and syntaxin 6 staining to visualize the Golgi apparatus, we observed that glutamine deprivation triggered Golgi–lysosome co-clustering (Fig. 3d; Supplementary Fig. 4A). Similar results were obtained in H4 and HCT116 cells (Supplementary Fig. 4B).

### mTORC1 regulates endomembrane reorganization

The rapamycin-sensitive mTORC1 kinase complex is generally considered to be a negative regulator of autophagy. In nutrient-replete cells, active mTORC1 suppresses early events in the autophagy pathway^35^,^36^. Conversely, nutrient starvation or treatment with mTOR inhibitors suppresses mTORC1 activity, leading to the de-repression of autophagy. To determine whether the autophagic response to glutamine restriction was attributable to attenuated mTORC1 activity, we examined phosphorylation of S6 kinase 1 (S6K1), a known mTORC1 substrate (Fig. 3e). In response to glutamine deprivation, U2OS cells reduced p62 levels, indicating increased autophagy (Fig. 2c). In contrast, S6K1 phosphorylation was unchanged in response to glutamine deprivation (Fig. 3e). Cells cultured in medium containing either no amino acids or only essential amino acids displayed nearly complete dephosphorylation of S6K1. These results indicate that, unlike more global amino acid starvation, removal of glutamine alone from the culture medium did not lead to a reduction in mTORC1 activity. Thus, glutamine deprivation-induced autophagy, unlike global amino-acid deprivation, triggers an autophagic response in the absence of reduced mTORC1 activity.

We next tested whether mTOR colocalized with the lysosomal cluster provoked by glutamine deprivation. Under nutrient-replete conditions, mTORC1 localizes to the lysosomal surface, where its activity is coupled to the extrusion of amino acids from the lysosomal lumen^37^,^38^. Immunofluorescence staining revealed mTOR colocalization with the lysosome cluster in glutamine-restricted cells, consistent with the retention of mTORC1 activity in these cells (Fig. 3f). In cells deprived of all amino acids, mTORC1 activity was completely suppressed and mTORC1-lysosome colocalization was abolished, in agreement with published reports (Fig. 3e,f)^37^. Complete amino-acid deprivation also led to lysosomal dispersion throughout the cytoplasm (Fig. 3f). Treatment of glutamine-starved cells with WYE-125132, a highly selective inhibitor of mTOR kinase activity^39^, blocked the formation of lysosomal clusters and elicited a dispersed pattern of LAMP1 staining similar to that observed on amino-acid starvation (Fig. 3f). However, a substantial level of mTOR-lysosome colocalization remained in the mTOR inhibitor-treated cells (Fig. 3f). These results suggested that lysosomal localization of mTORC1 was necessary but not sufficient to drive lysosomal clustering: mTORC1-dependent signalling was also required for this response. WYE-125132 exposure or amino-acid starvation also blocked phagophore clustering (Supplementary Fig. 5), indicating that the formation of these glutamine deprivation-induced endomembrane structures was dependent on mTOR kinase activity. These results suggested that glutamine deprivation leads to a cytoplasmic endomembrane reorganization response with parallels to the TASCC complex seen in cells undergoing OIS.

### IL-8 secretion is autophagy independent

**Figure 4:**
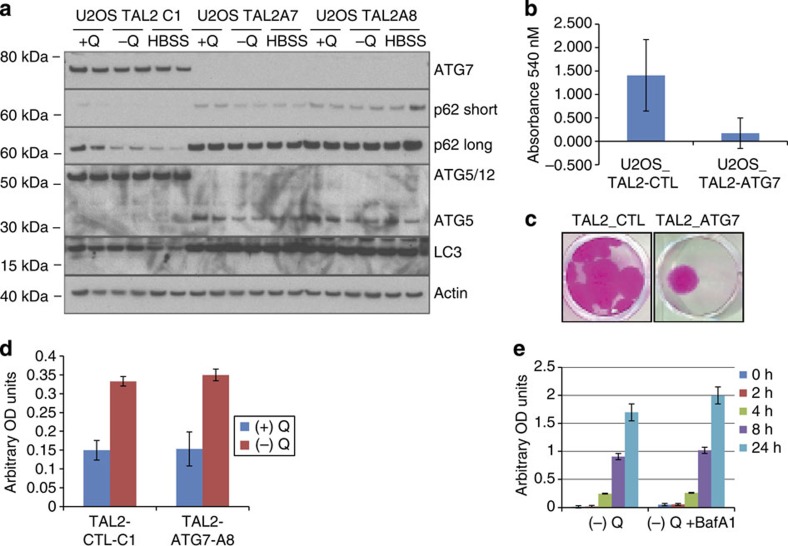
Autophagy is not required for glutamine deprivation-induced IL-8 secretion. (**a**) Immunoblot of autophagy-related proteins in U2OS cells expressing a control TALEN (U2OS-TAL2-C1) or two independent subcloned TALEN cell lines targeting ATG7 (U2OS-TAL2-A7, U2OS-TAL2-A8). p62 long and short refer to either long or short exposure times during film development. (**b**) U2OS cells expressing an ATG7-TALEN (TAL2-ATG7) or control TALEN (TAL2-CTL) were subjected to glutamine starvation for 6 days followed by 6 days recovery in glutamine-replete medium. Cells were then stained with SRB and absorbance was read at 540 nM. Graph shows average results from five clonal sub-lines for each condition. The statistical significance (*P* value) was determined by a two-tailed, paired Student’s *t*-test. *P*<0.05. (**c**) Representative images of SRB-stained clones in **b**. (**d**) IL-8 enzyme-linked immunosorbent assay (ELISA) of U2OS-TAL2-CTL-C1 (control) and U2OS-TAL2-ATG7-A8 (ATG7 knockout) following 24 h glutamine deprivation. (**e**) IL-8 ELISA of U2OS parental cells starved of glutamine for the indicated times with or without BafA1. Error bars in all figures represent s.d. of three biological replicates. OD, optical density.

The TASCC was shown to couple autophagic activity to IL-8 mRNA translation and IL-8 secretion^23^. We therefore sought to test whether a similar relationship between autophagy and chemokine production existed in glutamine-restricted cancer cells. U2OS cells were transfected with transcription activator-like effector nucleases (TALENs) targeting the essential autophagy gene *ATG7*. Clonal U2OS cell lines transfected with the ATG7-TALEN (U2OS-TAL2-A7 or U2OS-TAL2-A8) expressed no detectable ATG7 protein (Fig. 4a). ATG7 is an E1-like enzyme that is required for autophagy due to its roles in the conjugation of ATG12 to ATG5, and of ATG8 (LC3) to phosphatidylethanolamine^40^. Relative to U2OS cells transfected with the control TALEN (U2OS-TAL2-C1), the ATG7-TALEN clones expressed no detectable ATG5/12 conjugates, and displayed a marked accumulation of p62 in complete or amino-acid-deficient medium, consistent with defective basal and inducible autophagy (Fig. 4a). As predicted, the absence of autophagy in the U2OS-TAL2 clones led to reduced survival in response to long-term (6 days) culture in glutamine-free medium (Fig. 4b,c).

The U2OS-TAL2 clones were then subjected to the standard 24-h glutamine deprivation protocol, followed by determinations of IL-8 secretion. Contrary to our expectation, these autophagy-defective cells were fully competent to express and secrete IL-8 in response to glutamine restriction (Fig. 4d). Similar results were observed in A549 cells transfected with the ATG7-targeted TALEN (data not shown). In addition, IL-8 production was not affected by treatment of glutamine-deprived U2OS cells with BafA1, a pharmacological inhibitor of autophagy (Fig. 4e). Taken together, these data indicate that, although autophagy is required for optimal cell survival during extended glutamine deprivation, the expression and secretion of IL-8 induced by short-term glutamine withdrawal is independent of autophagic activity.

### mTORC1-JNK signalling stimulates IL-8 secretion

**Figure 5:**
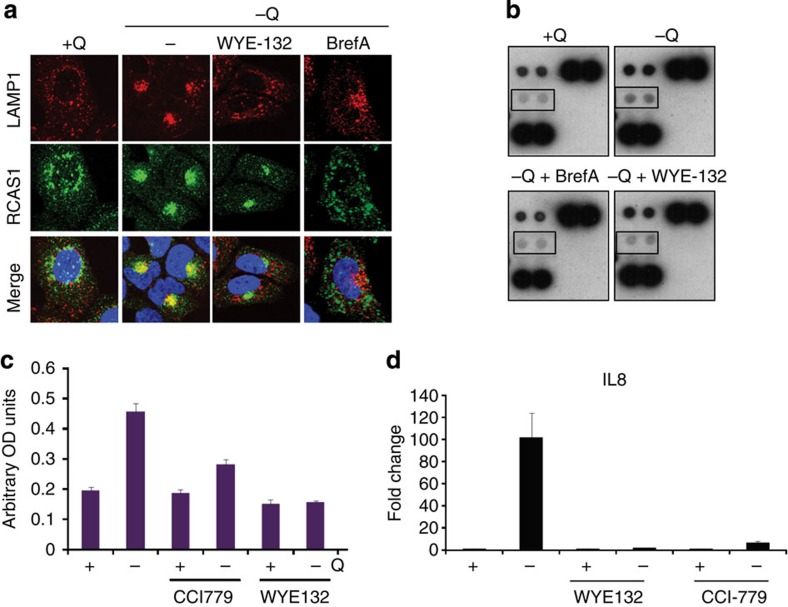
Glutamine deprivation-induced IL-8 secretion requires mTOR activity. (**a**) Immunofluorescence images of U2OS cells stained for LAMP1 and the Golgi marker RCAS1 after 24 h glutamine deprivation with or without treatment with 1 μM BrefA or 100 nM WYE-125132. Scale bar, 15 μm. (**b**) Cytokine array analysis of conditioned medium from U2OS cells subjected to glutamine deprivation in the presence of 1 μM BrefA or 100 nM WYE-125132. (**c**) IL-8 enzyme-linked immunosorbent assay in U2OS cells after 24 h glutamine deprivation with or without CCI-779 or WYE-125132. (**d**) RT–PCR analysis of IL-8 mRNA expression in U2OS cells after 24 h glutamine deprivation with or without CCI-779 or WYE-125132. Error bars in all figures represent s.d. of three biological replicates. OD, optical density.

To better understand the mechanisms regulating glutamine deprivation-induced IL-8 secretion, we examined the possible role of mTORC1 in this response by treating glutamine-deprived U2OS cells with WYE-125132. WYE-125132 blocked the formation of the Golgi body–autophagy–lysosome cluster described above (Fig. 5a) and strongly reduced IL-8 secretion in the glutamine-deprived cells (Fig. 5b,c). Disruption of the Golgi apparatus by brefeldin-A inhibited both the colocalization of Golgi endomembranes with lysosomes (Fig. 5a) and IL-8 secretion during glutamine restriction (Fig. 5b). Collectively, these data indicate that mTOR kinase activity is required for the juxtapositioning of the Golgi apparatus, autophagy components and mTORC1-associated lysosomes in glutamine-restricted cells. Moreover, both an intact Golgi apparatus and CCI-779-sensitive mTORC1 activity (Fig. 5b,c) were required for the production of IL-8 by glutamine-restricted cells. An unanticipated observation was that mTORC1 inhibition by WYE-125132 or CCI-779 blocked IL-8 induction at the mRNA level, suggesting that an mTORC1-dependent mechanism was driving *IL8* gene transcription in these cells (Fig. 5d).

**Figure 6:**
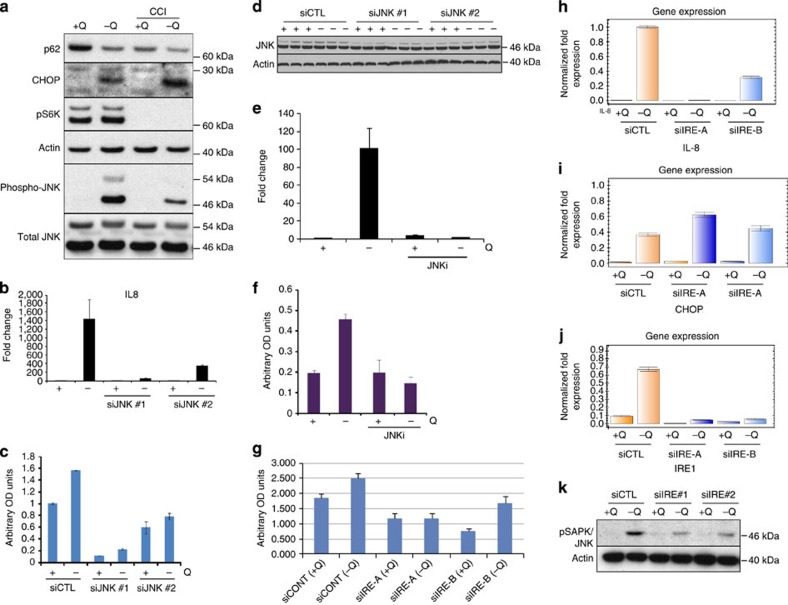
mTORC1 regulates ER stress-induced JNK signalling. (**a**) Immunoblot analysis of autophagy, ER stress- and mTOR-related proteins, and JNK in U2OS cells after 24 h glutamine deprivation with or without CCI-779. (**b**) RT–PCR analysis of IL-8 mRNA expression in U2OS cells transfected with two different siRNAs targeting JNK (JNK #1 and JNK #2) and subjected to 24 h glutamine deprivation. (**c**) IL-8 enzyme-linked immunosorbent assay (ELISA) of conditioned media from the experiment described in **b**. (**d**) Immunoblot analysis of JNK protein expression in JNK siRNA-treated cells. (**e**) RT–PCR analysis of IL-8 expression in U2OS cells after 24 h glutamine deprivation with or without the JNK inhibitor SP600125 (JNKi). (**f**) IL-8 ELISA of conditioned medium from the experiment described in **e**. (**g**) IL-8 ELISA of U2OS cells transfected with two different siRNAs targeting IRE1 (IRE-A and IRE-B) and subjected to glutamine deprivation for 24 h. (**h**) RT–PCR analysis of IL-8 mRNA expression from experiment described in **g**. (**i**) RT–PCR analysis of CHOP mRNA expression from experiment described in **g**. (**j**) RT–PCR analysis of IRE1 mRNA expression from experiment described in **g**. (**k**) Immunoblot analysis of experiment described in **g** showing phosphorylation of JNK. Error bars in all figures represent s.d. of three biological replicates. OD, optical density.

The c-Jun NH2-terminal Kinase (JNK) is a stress-activated protein kinase that is activated in response to ER stress^41^ and is a known upstream activator of IL-8 (ref. 42)^42^. JNK was strongly phosphorylated following 24 h glutamine deprivation. Inhibition of mTORC1 activity with CCI-779 partially blocked JNK phosphorylation. Suppression of mTORC1 did not simply lead to a global shutdown of cellular translation since CHOP protein induction was unaffected by CCI-779 (Fig. 6a). Suppression of JNK with small interfering RNA (siRNA) blunted IL-8 transcription and secretion (Fig. 6b–d). In addition, treatment with a JNK kinase inhibitor strongly inhibited glutamine deprivation-induced IL-8 transcription and secretion, providing strong evidence for the role of JNK in glutamine deprivation-induced IL-8 secretion (Fig. 6e,f).

mTORC1 was previously reported to play an important role in the ER stress-induced IRE1-JNK pathway^41^, prompting us to examine the role of IRE1 in glutamine deprivation-induced IL-8 secretion. Knockdown of IRE1 with siRNAs (Fig. 6j) blocked both IL-8 transcription and secretion following glutamine deprivation (Fig. 6g,h). In contrast, IRE1 siRNA did not affect induction of CHOP (Fig. 6i). IRE1 siRNA also strongly inhibited glutamine deprivation-induced JNK phosphorylation (Fig. 6k), implying that the IRE1-JNK arm of ER stress was specifically required for glutamine deprivation-induced IL-8 secretion.

We next tested whether the ER stress inducer TPG also induced IL-8 secretion via activation of the same pathway. U2OS cells were treated with TPG with or without CCI-779 or WYE-125132. TPG induced IL-8 transcription and secretion in an mTOR-dependent manner (Supplementary Fig. 6A–C). TPG also induced CHOP transcription in a mTORC1-independent fashion, similar to the response induced by glutamine deprivation (Supplementary Fig. 6D). Notably, TPG-induced JNK phosphorylation was also suppressed by mTOR inhibition (Supplementary Fig. 6E).

### IL-8 secretion is a response to deficient anaplerosis

**Figure 7:**
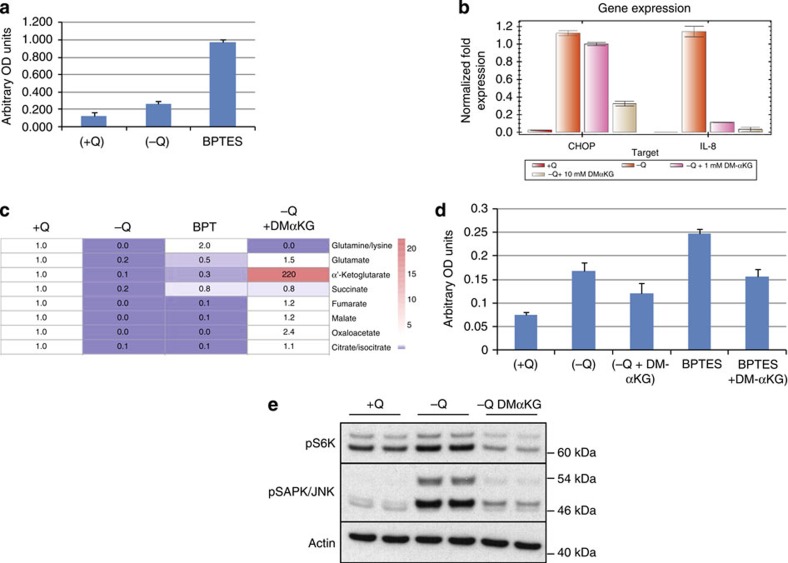
Glutamine starvation and glutamine metabolism inhibitors induce IL-8 secretion that is reversed by α-KG supplementation. (**a**) IL-8 enzyme-linked immunosorbent assay (ELISA) on U2OS cells starved of glutamine or treated with BPTES (10 μM) for 24 h. (**b**) RT–PCR analysis of IL-8 and CHOP mRNA expression in U2OS cells starved of glutamine for 24 h and co-treated with DMαKG. (**c**) Metabolite analysis of U2OS cells starved of glutamine, treated with BPTES or glutamine-starved co-treated with DMαKG. (**d**) IL-8 ELISA of U2OS cells starved of glutamine or treated with BPTES for 24 h and co-treated with DMαKG. (**e**) Immunoblot analysis of pS6K and pJNK in U2OS cells starved of glutamine for 24 h and co-treated with DMαKG. Error bars in all figures represent s.d. of three biological replicates. OD, optical density.

Finally, we tested the effects of a glutamine metabolism inhibitor on IL-8 secretion. Bis-2-(5-phenylacetamido-1,3,4-thiadiazol-2-yl)ethyl sulfide (BPTES) is a selective inhibitor of the kidney-type glutaminase isoform (GLS1), which catalyses the deamination of glutamine to glutamate. BPTES strongly induced both IL-8 and CHOP, suggesting that BPTES treatment phenocopies the effects of glutamine deprivation on these stress responses (Fig. 7a,b). Metabolomic analyses revealed that both glutamine deprivation and BPTES treatment depleted the TCA cycle intermediates α-KG, citrate, succinate and fumarate. BPTES treatment also led to an increase in intracellular glutamine pools, suggesting that defective glutamine metabolism, rather than depletion of intracellular glutamine levels, was responsible for the sequence of events leading to IL-8 induction (Fig. 7c). To test this idea, a cell-permeable form of α-KG, the TCA cycle intermediate produced by glutamine deamination, was examined for its ability to reverse the stress-response mechanism that drives IL-8 secretion. Treatment with dimethyl alpha-KG (DM-αKG) successfully restored cellular TCA cycle pools to levels comparable to those seen in the glutamine-replete condition (Fig. 7c). Importantly, DM-αKG treatment reduced IL-8 transcription and secretion in both glutamine-restricted (Fig. 7b,d) and BPTES-treated cells (Fig. 7d). Finally, glutamine deprivation-induced JNK phosphorylation was also blocked by the addition of DM-αKG (Fig. 7e). Together, these results indicate that the impairment of TCA cycle function during glutamine restriction is partially responsible for increases in IL-8 transcription and secretion observed in these studies.

## Discussion

Glutamine has emerged as both an important fuel for anabolic metabolism and a regulator of key cellular responses, such as growth factor receptor expression^12^, mTORC1 activation^43^,^44^ and autophagy^20^,^21^. The present findings shed further light on the complex interplay between glutamine and cell physiology by demonstrating that a relatively brief period (24 h) of glutamine restriction leads to a striking reorganization of several cytoplasmic organelles, accompanied by stimulation of autophagy and an autophagy-independent stress response resulting in the induction of IL-8 expression and secretion. The IL-8 release provoked by glutamine restriction was dependent on the activity of a mTORC1-IRE1-JNK pathway that triggered both transcription and secretion of this chemokine. Given the pleiotropic actions of IL-8 in the tumour microenvironment, the present findings have significant implications for both tumour pathophysiology and therapeutic strategies aimed at interfering with glutamine uptake and metabolism in cancer patients^45^.

The persistence of mTORC1 activity in glutamine-deprived cells was unexpected, given that amino acid availability is a well-established regulator of mTORC1 activity, and glutamine in particular had been causally linked to amino-acid-dependent mTORC1 activation^43^,^44^. The specific assay conditions used in our study provide a plausible explanation for the maintenance of mTORC1 activity in glutamine-deprived cells. Our experimental protocol involved a relatively brief (24 h) exposure of cells to standard, serum-containing culture medium lacking only glutamine. In contrast, the earlier reports implicating glutamine in amino-acid-dependent mTORC1 activation employed a distinct protocol involving an initial period of complete amino-acid starvation followed by re-addition of specific amino acids and subsequent assessment of mTORC1 activity. Cells treated under these conditions were dependent on glutamine and leucine for mTORC1 reactivation after amino-acid starvation^43^,^44^. Glutamine-induced mTORC1 activation in these studies involved at least two mechanisms, one related to glutamine-dependent import of the mTORC1-activating amino acid, leucine, and the other via the production of α-KG. The distinct glutamine restriction protocol used in our studies permitted examination of glutamine-related phenotypic responses in the absence of confounding inhibitory effects on mTORC1 function.

The importance of persistent mTORC1 activity in glutamine-deprived cells was highlighted by our findings that both the endomembrane reorganization and autophagic responses induced by glutamine restriction were strongly suppressed by pharmacologic inhibitors of mTORC1 signalling functions. The endomembrane reorganization induced by glutamine deprivation involved juxtapositioning of the Golgi with phagophores, autophagosomes and lysosomes in a single cytoplasmic cluster. Relocalization of cytoplasmic organelles was not a global response, in that the distribution of mitochondria in the cytoplasm was not altered by glutamine starvation (results not shown). Immunofluoresence microscopy revealed the presence of mTOR in the lysosomal clusters, consistent with recent reports indicating that active mTORC1 is associated with the lysosomal compartment^37^,^38^. Inhibition of mTORC1 activity with the rapalogue, CCI-779 or the mTOR kinase inhibitor, WYE-125132, stimulated autophagy, but did not lead to endomembrane clustering. Indeed, treatment of glutamine-deprived cells with WYE-125132 blocked the appearance of the endomembrane cluster, indicating that the relocalization and/or stability of this structure were dependent on mTORC1 signalling, presumably on the lysosomes.

The spatial colocalization of the autophagy machinery with lysosomes appeared to drive rapid, efficient fusion of mature autophagosomes with lysosomes, as indicated by our inability to visualize GFP-LC3-labelled autophagosomes in these clusters in the absence of the lysosomal inhibitor, BafA1. We propose a bidirectional interplay between mTORC1 and the endomembrane clusters in which autophagy fuels the lysosomal lumen with amino acids that stimulate mTORC1 activity, and mTORC1 in turn drives events that support the assembly and/or maintenance of these clusters. The concentration of mTORC1 complexes near the Golgi apparatus might also facilitate the translation of mRNAs in nearby rough ER sites. This model is reminiscent of recent findings demonstrating that autophagy supports mTORC1 activation during nutrient starvation and that active mTORC1 is required for the reformation of lysosomes from autolysosomes^46^. Perhaps, the lysosomal clustering observed in glutamine-deprived cells reflects, at least in part, the localized regeneration of lysosomes in a subcellular compartment with intense autophagic activity.

The endomembrane redistribution elicited by glutamine withdrawal also shares features with the previously reported TASCC in cells undergoing OIS^23^. In this study, fibroblasts induced to undergo senescence by acute expression of the H-Ras oncoprotein displayed autophagosome–lysosome clusters and concomitant activation of autophagy and mTORC1. How might seemingly disparate, stress-inducing events, such as glutamine restriction and oncoprotein expression, trigger the formation of similar autophagy-lysosome clusters? A reasonable hypothesis is that glutamine deprivation and HRAS expression converge on a common stress-response network provoked by the accumulation of unfolded proteins in the ER (Table 1; Fig. 1; ref. 23). The ER stress response is activated in response to a broad range of stimuli, and results in global translational suppression, accompanied by the induced expression of a gene set whose encoded proteins act to restore ER homeostasis and preserve cell viability, over the short term, and to trigger apoptotic cell death in the setting of chronic ER stress^24^. Our studies indicate that the ER stress response invoked by glutamine restriction leads to the expression of genes associated with autophagy and the SASP.

The relationship between autophagic activity and IL-8 secretion was the most critical difference between OIS-induced IL-8 (ref. 23)^23^ and the chemokine secretion response evoked by glutamine deprivation in the present study. Autophagic activity was causally related to OIS-mediated chemokine production, whereas the IL-8 expression induced by glutamine restriction was maintained in cells in which the autophagy pathway was genetically disrupted. Taken together, the earlier and present findings suggest that OIS and glutamine deprivation induce a similar clustering of a specific subset of cytoplasmic organelles and concomitant increases in autophagic flux. Although both stress-induced pathways converge on the production of IL-8 and other chemokines, the contribution of autophagy to this shared downstream response is fundamentally different in cell subjected to OIS versus glutamine restriction.

Previous reports suggested that mTORC1 activity was required for induction of the ER stress-stimulated IRE1-JNK pathway, but was not involved in the activation of the parallel PERK and ATF6 pathways triggered by ER stress^41^. For example, rapamycin selectively suppressed IRE1-JNK signalling and attenuated ER stress-induced apoptosis in rat renal tubular epithelial cell lines^41^. Our results indicate that glutamine deprivation strongly activates JNK, and that this event is dependent on the upstream activities of mTORC1 and IRE1. Taken together, these data support a working model, which posits that glutamine deprivation provokes an IRE-1- and mTORC1-dependent increase in JNK activity that, in turn, leads to IL-8 expression and secretion. The precise mechanism by which glutamine restriction leads to chemokine secretion is incompletely understood. However, the observations that glutaminase inhibition phenocopied the effects of glutamine withdrawal, and the reversal of these responses by the glutamine deamination product, α-KG, argue that impaired TCA cycle activity underlies this multi-factorial stress response. One possible signal emanating from the loss of TCA cycle intermediates may be enhanced production of reactive oxygen species (ROS). Initial studies indicate that glutamine deprivation, but not leucine deprivation, results in robust ROS production, and the use of the ROS scavenger, *N*-acetylcysteine, partially blunts IL-8 secretion, suggesting a role for oxidative stress in the observed response (data not shown). The differential induction of IL-8 during glutamine versus leucine starvation argues that the secretory response is not simply a general, nonspecific outcome of amino-acid deprivation.

The present findings have potential implications for therapeutic strategies aimed at disrupting glutamine uptake and/or metabolism in cancer cells^16^. Certain cancer cells, such as those transformed by the Myc oncoprotein, are glutamine auxotrophs, and undergo cell death in the setting of glutamine withdrawal or in response to glutaminolysis inhibitors^13^,^14^. A recent report indicated that *MYCN*-amplified neuroblastoma cells undergo apoptotic death due to the activation of a nutrient stress-response pathway leading to accumulation of the pro-apoptotic PUMA and NOXA proteins^13^. The GLS inhibitor BPTES and its analogues have shown interesting preclinical activities and are currently in early clinical development^47^. Hence, our observation that BPTES triggers IL-8 secretion in cultured cells may be clinically relevant. If a similar response was invoked in tumour tissue during therapeutic inhibition of glutamine metabolism, the secretion of autocrine/paracrine-acting IL-8 might have significant effects on tumour pathophysiology and the tumour–host interface, which could lead to changes in therapeutic responsiveness. Previous reports demonstrated that autocrine IL-8 signalling stimulates the outgrowth of highly tumorigenic subpopulations that exhibit increased drug resistance and metastatic behaviour, and that paracrine IL-8 signalling promotes angiogenesis and an inflammatory microenvironment^48^. Additional studies are clearly needed to more fully understand the impact of the chemokine-secretory response on clinical outcomes in patients receiving drugs that interfere with glutamine metabolism, as well as the potential benefits of combining these agents with antagonists of IL-8 and other tumour-derived chemokines.

## Methods

### Antibodies and chemicals

The following antibodies were used for western blotting and immunofluorescence studies: LC3 (NB100-2220, Novus Biologicals, dilution 1:1,000), p62 (BD Transduction, catalogue #610832, dilution 1:1,000), actin (Sigma, A1978, dilution 1:100,000) and LAMP1 (ab25630, Abcam, dilution 1:100). Antibodies directed against GFP (#2555, dilution 1:100), phospho-S6K1 (p70 S6 Kinase Thr-389, #9205, dilution 1:1,000), S6K1 (p70 S6 Kinase, #2708, dilution 1:1,000), mTOR (#2983, dilution 1:100), RCAS1 (#6960, dilution 1:100), syntaxin 6 (#28690, dilution 1:100), CHOP (#2895, dilution 1:1,000), phospho-EIF2α Ser-51 (#9721, dilution 1:1,000), EIF2α (#2103, dilution 1:1,000), phospho-SAPK/JNK, Thr183/Tyr185 (#9255, dilution 1:1,000) and JNK1 (#3708, dilution 1:1,000) were obtained from Cell Signaling Technologies.

Bafilomycin A1 (catalogue #B1793), tunicamycin (catalogue #T7765), thapsigargin (catalogue #T9063), brefeldin-A (catalogue #B7651), dimethyl-α-ketoglutarate (catalogue #349631), BPTES (catalogue #SML0601) and DON (6-diazo-5-oxo-L-norleucine; catalogue #D2141) were obtained from Sigma-Aldrich. The JNK inhibitor SP600125 was obtained from R&D Systems (catalogue #1496). CCI-779 and WYE-125132 were synthesized at Pfizer, and have been described previously^29^,^39^. Lysotracker Green DND-26 (#L-7526) and Red DND-99 (#L7528) were obtained from Invitrogen Technologies. All chemicals and kits were used according to the manufacturers’ recommendations, unless otherwise indicated.

### Cell culture

All cell lines used in the study were grown in a humidified 5% CO_2_ atmosphere. U2OS, H4, HCT116 and A549 cell lines were purchased from the ATCC and iBMK (generously provided from the laboratory of Dr Eileen White) cells were cultured in DMEM (Gibco, catalogue #11995), containing 2 mM glutamine, supplemented with 10% fetal bovine serum (FBS) and 1% penicillin–streptomycin (complete DMEM). The iBMK cell lines 6.1B11 (*atg5*^+/+^) and 7.1B4 (*atg5*^*−*/−^) and U2OS GFP-LC3 cells have been described before^20^,^32^. U2OS mCherry-ATG5 cells were generated by transfecting early passage U2OS cells with mCherry-ATG5 (EX-M0036-M55 from Genecopoeia) and selecting for G418 resistance (500 μg ml^−1^). U2OS mCherry-ULK1 cells were generated by transfecting U2OS cells with mCherry-ULK1 (EX-M0809-Lv111 from Genecopoeia) and selecting for puromycin resistance (1 μg ml^−1^). U2OS mCherry-ATG5 cells were subsequently transfected with GFP-LC3 to generate U2OS mCherry-ATG5/GFP-LC3 cells. U2OS mCherry-GFP-LC3 cells were generated by transducing U2OS cells with a mCherry-GFP-LC3 plasmid.

For glutamine deprivation, cells were plated overnight in complete DMEM, briefly washed with phosphate-buffered saline (PBS) and then transferred into glutamine-free medium (glutamine- and pyruvate-free DMEM (Cellgrow, catalogue #15-017-CV)) supplemented with 10% dialyzed FBS (Gibco, catalogue #26400) and 1 mM sodium pyruvate (Cellgro, catalogue #25-000-CI). The corresponding glutamine-replete medium was prepared by addition of 2 mM glutamine (Cellgro, catalogue #25-005-CV) to glutamine-free medium. Cells were cultured for 24 h in glutamine-free medium, unless otherwise indicated. For leucine deprivation, cells were plated in glutamine/leucine-free media (MP Biomedicals, #1342149) supplemented with 10% dialyzed FBS, sodium pyruvate and either 2 mM glutamine or 0.8 mM leucine.

### Immunoblotting

For immunoblotting experiments, 500,000 cells were plated overnight in six-well tissue culture plates. After treatment for the indicated times, cells were washed once with PBS, and cell lysates were prepared by scraping in NuPAGE lithium dodecyl sulfate (LDS) buffer (Invitrogen, catalogue #NP0008), followed by sonication in a water bath. Protein concentrations were determined with the Bio-Rad RC/DC protein assay, and equal amounts of protein were electrophoresed through NuPAGE 4–12% bis–tris gradient gels with 2-(N-morpholino)ethanesulphonic acid (MES) running buffer. Proteins were transferred to nitrocellulose and incubated with primary antibody overnight at 4 °C. Proteins were detected using chemiluminescence with appropriate horseradish peroxidase-conjugated secondary antibodies. Uncropped scans of the immunoblot membranes are provided as Supplementary Figs 7–14.

### Live cell microscopy

Live U2OS cells expressing epitope-tagged proteins were imaged with an inverted Olympus IX51 fluorescent microscope at × 20 magnification. For quantification of GFP-LC3 puncta, cells displaying >10 brightly fluorescent GFP-LC3 puncta were counted as positive. For phagophore–lysosome colocalization studies, treated cells were co-stained with Lysotracker Green for lysosomes and Hoechst-33342 for nuclei. For mCherry-GFP-LC3 cells, simultaneous images in the Green and Red channel were acquired for the same field of cells followed by evaluation of the digitally merged image to estimate autophagosome and autolysosome number.

### Immunofluorescence analysis

U2OS cells were grown overnight on borosilicate glass chambered slides (Nunc, catalogue #155409). At the end of the experiment, cells were fixed and permeabilized with the Fix and Perm kit (GAS004, Invitrogen). Immunostaining was then performed by overnight incubation with the appropriate antibodies (1:100 dilutions) at 4 °C. The cells were then stained with fluorescein isothiocyanate- or Cy3-conjugated secondary antibodies, together with 4′,6-diamidino-2-phenylindole (DAPI) as a nuclear counterstain. Confocal images were generated using an LSM 510 Meta microscope at × 40 magnification.

### Microarray analysis

Microarray analysis was performed as described^49^. Briefly, 2 million U2OS cells were cultured for 24 h in glutamine-free medium or the same medium with added glutamine. Cells were collected in buffer RLT (Qiagen) followed by RNA isolation using the RNeasy kit (#74104, Qiagen). Complementary DNA synthesis and subsequent *in vitro* antisense RNA (cRNA) amplification and biotin labelling were performed as described^49^. For each sample, 10 μg of biotin-labelled cRNA was fragmented and hybridized to Human Genome U133+2 GeneChip oligonucleotide arrays (Affymetrix) using buffers and conditions recommended by the manufacturer. GeneChips were then washed and stained with Streptavidin R-phycoerythrin (Molecular Probes) using the GeneChip Fluidics Station 450, and scanned with a Affymetrix GeneChip Scanner 3000. Microarray data was converted into MIAME format and was uploaded into the Gene Expression Omnibus (GEO) database with accession number GSE59931.

### Real-time PCR analysis

U2OS cells (500,000) were plated overnight followed by treatment for 24 h with glutamine-free medium, glutamine-free medium with added glutamine or with glutamine-containing medium supplemented with 1 μM TPG. Cells were collected in buffer RLT (Qiagen) followed by RNA isolation using the RNeasy kit (#74104, Qiagen). cDNA was prepared with the iScript cDNA synthesis kit (170–8890, Bio-Rad) according to the manufacturer’s instructions. Real-time PCR analysis was then performed with standard cycling conditions on a Bio-Rad CFX96 real-time system using TaqMan primer sets offering the best coverage. Relative transcript levels were calculated using the comparative *C*_t_ method and normalized to the housekeeping gene, beta-microglobulin.

### Cytokine array

Cytokine profiling was performed with the Human Cytokine Array Kit, Panel A (#ARY005, R&D Systems). Briefly, conditioned medium from cells treated for 24 h with glutamine-free or -supplemented medium was collected and clarified by centrifugation. The cleared medium was diluted, mixed with a biotinylated detection antibody cocktail and then incubated with the Human Cytokine Array Panel A membranes. Following a wash to remove unbound material, streptavidin-horseradish peroxidase and chemiluminescent detection reagents were added sequentially and the cytokine array was visualized by autoradiography.

### IL-8 enzyme-linked immunosorbent assay

IL-8 levels in the cell culture supernatants were measured with a quantitative sandwich enzyme immunoassay (Quantikine ELISA Kit—D8000C, R&D Systems). Briefly, U2OS or A549 cells were plated (5 × 10^5^ cells per well) in a six-well plate and were allowed to adhere overnight. Cells were then treated for 24 h with glutamine-free or -supplemented medium. Conditioned media were collected for determination of IL-8 levels with the Quantikine ELISA kit.

### siRNA transfections

U2OS cells were plated overnight in tissue culture-treated, six-well plates (3 × 10^5^ cells per well), and were subsequently transfected with 50 nM siRNA per well mixed with Lipofectamine RNAiMax (13778-150, Invitrogen) according to the manufacturer’s protocol. Cells were subjected to glutamine deprivation at 72 h post transfection. The following siRNA duplexes were used and purchased from Ambion: control siRNA (#4390843), siJNK#1 (S28271), siJNK#2 (S28272), siIRE1#1 (S200430, S200432) and siIRE1#2 (S20800, S20801).

### ATG7 TALENS

ATG7 TALENS were purchased from PNA Bio. U2OS cells were plated at a density of 2 × 10^6^ in 10 cm plates. After 24 h, cells were transfected with cytomegalovirus (CMV) TALEN_L2 (5′-TATTGGAACACTGTATAACA-3′), CMV TALEN_R2 (5′-TGTCCTTGGGAGCTTCATCC-3′) and RG2S_2_CMV (GFP/red fluorescent protein surrogate reporter) using Lipofectamine 2000 at a ratio of 1:1:2. At 48 h post transfection, single cells were sorted into individual wells of a 96-well plate. Control cell lines were generated from cells expressing one TALEN module (which results in red fluorescence) by fluorescence-activated cell sorting-based sorting for red fluorescent protein-only expressing cells (U2OS_TAL2_CTL). ATG7-knockout cells were generated from cells expressing both the left and right TALEN modules, which result in both red and green fluorescence (U2OS_TAL2_A7, U2OS_TAL2_A8). For experiments, U2OS_TAL2_CTL and U2OS TAL2_A8 were used unless otherwise indicated.

### Sulphorhodamine B viability assay

Cells were plated at a density of 100,000 cells per well in a 24-well plate. After 24 h, the medium was changed as described in the Results section, and cells were incubated for 6 days. The culture medium was replaced with complete medium and cells were allowed to recover for 6 days. Following recovery, cells were fixed in 10% trichloroacetic acid for 1 h at 4 °C. Cells were washed five times in water and stained with a 0.057% (w/v) solution of SRB (Sulphorhodamine B; Sigma, catalogue #S-9012) in 1% acetic acid. The plate was then washed five times with 1% acetic acid. SRB was solubilized in 10 mM unbuffered Tris base and absorbance was read at 540 nm.

### Metabolic analyses

Cells were seeded in triplicate in six-well plates. After the indicated treatments, the cell culture medium was removed from plates and cells were washed three times with cold PBS. The cell culture plates were stored at −80 °C until analysis. Intracellular metabolites were extracted by adding 0.25 ml of cold organic extraction buffer consisting of 40/40/20 mixture of acetonitrile/methanol/water for 15 min on ice followed by addition of 0.25 ml water for 5 min on ice. Ten μl of 2 μM iso-ATP and 5-fluoro-2-methylpyridine (injection standards) was added to the pooled metabolites, and the resulting solutions were centrifuged at 16,000 *g* at 4 °C for 15 min. Two Liquid Chromatography-Multiple Reaction Monitoring (LC-MRM) methods were used for analysis of the indicated metabolites: (1) an Imtakt Unison UK-Phenyl 75 × 2.0 mm column coupled with AB SCIEX API4000 for monitoring TCA metabolites in the negative ionization mode, with mobile phase A of 100% water containing 0.1% formic acid and mobile phase B of 100% acetonitrile containing 0.1% formic acid, was used at flow rate of 0.3 ml min^−1^ and column oven temperature of 30 °C. The following gradient was used: 0–4 min 0% solvent B, 4–5 min 0–100% solvent B, 5–8 min 100% solvent B, 8–9 min 100–0% B and 9–15 min 0% solvent B. (2) An Imtakt Scherzo SM-C18 150 × 2.0 mm column coupled with AB SCIEX API4000 for monitoring amino acids in the positive ionization mode, with mobile phase A of 100% water containing 0.1% formic acid and mobile phase B of 100% acetonitrile containing 0.1% formic acid, was used with the flow rate of 0.3 ml min^−1^ at 30 °C. The following gradient was used: 0–6 min 0% solvent B, 6–16 min 50% solvent B, 16–18 min 50–100% solvent B, 18–20 min 100% solvent B, 20–20.5 min 100–0% solvent B and 20.5–30 min 0% solvent B.

### Statistics

All experiments presented were performed in triplicate. Error bars on graphs represent s.d. unless otherwise stated. *P* values were calculated using a paired student’s *t*-test, two tailed, type 1.

## Author contributions

N.P.S. and K.B. designed and performed all the experiments in the paper. C.H.E. and V.R.F. provided valuable insight and guidance. F.W. and J.M. performed the metabolomics analysis. M.F. performed the microarray analysis and R.T.A. supervised all experiments and edited the final manuscript.

## Additional information

**How to cite this article:** Shanware, N. P. *et al.* Glutamine deprivation stimulates mTOR-JNK-dependent chemokine secretion. *Nat. Commun.* 5:4900 doi: 10.1038/ncomms5900 (2014).

## Supplementary Material

Supplementary Information Supplementary Figures 1-14

## Figures and Tables

**Table 1:** Gene expression changes following glutamine deprivation.

**Gene name**	**Fold change**	***P*** **value**
*Autophagy related*		
LC3 (MAP1LC3B)	4.8	0.0000
p62 (SQSTM1)	2.2	0.0001
WIPI1	3.7	0.0003
UVRAG	2.2	0.0043
*Lysosome related*		
ATP6V1C1	1.4	0.0026
ATP6V0A2	4.0	0.0004
ATP6V1D	1.6	0.0043
*ER stress related*		
DDIT3 (CHOP)	17.2	0.0022
XBP1	1.3	0.0941
PPP1R15A (GADD34)	21.5	0.0000
ATF6	1.5	0.0405
CCND1 (cyclin D1)	−5.7	0.0024
*SA-secretory proteome*		
IL-6	1.9	0.0076
IL-8	8.7	0.0325
CCL20 (MIP-3A)	5.9	0.0087
CCL3 (MIP-1A)	2.8	0.0383
Amphiregulin (Areg)	1.6	0.0475
Epiregulin (Ereg)	6.0	0.0082
bFGF (FGF2)	2.8	0.0022
KGF (FGF7)	4.7	0.0087
VEGF	2.5	0.0029
NGFB	7.0	0.0108
MMP1	3.3	0.0148
MMP3	2.3	0.0013
PAl-1 (SERPINE1)	2.6	0.0179
EGFR	5.1	0.0122

IL, interleukin.

U2OS cells were cultured for 24 h in the presence or absence of glutamine, and gene microarray analysis was performed. Significantly altered genes were grouped into the pathways specified in the table. Numbers are derived from three replicate samples for each condition. The column labelled fold change indicates the averaged ratio of expression in glutamine-restricted versus the glutamine-replete cultures. The statistical significance (*P* value) was determined by a two-tailed, paired Student’s *t*-test.
