# Supplementary material for: Glutamine deprivation stimulates mTOR-JNK-dependent chemokine secretion
Source: Nat Commun. 2014 Sep 25;5:4900. doi: 10.1038/ncomms5900 (PMC4200525; doi:10.1038/ncomms5900)
Supplement: Supplementary Information — Supplementary Figures 1-14 [file ncomms5900-s1.pdf]

Supplementary Figures

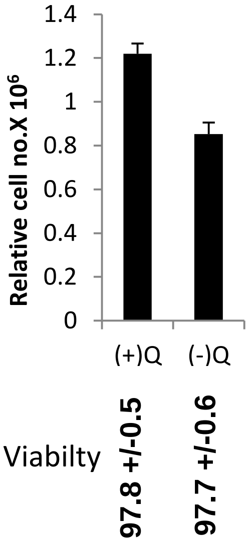

Supplementary Figure 1. Effect of 24 h glutamine restriction on cell growth.

U2OS cells were starved of glutamine 24 h. Bars display relative cell number.Error bars represent standard deviation of three biological replicates.

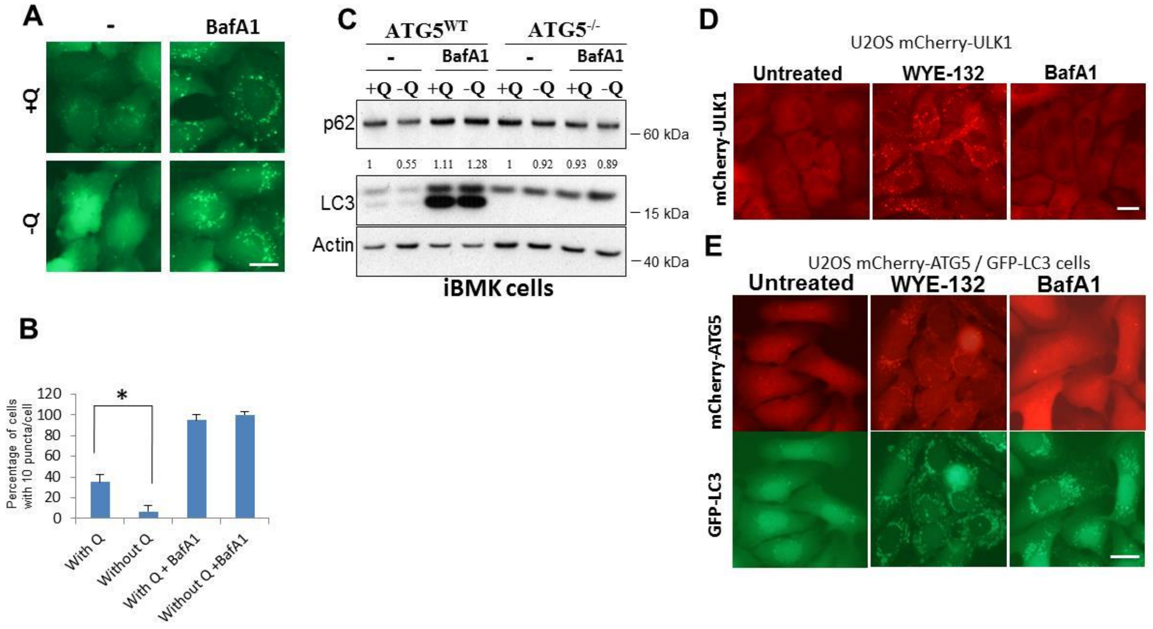

Supplementary Figure 2. Glutamine deprivation induces autophagic flux.

(A) Microscopic images of U2OS-GFP-LC3 cells following 8 h glutamine starvation with or without BafA1. Scale bar = 10 μm. **(B)** Graphical summary of experiments performed as described in panel A. Percentage of cells with greater than 10 puncta per cell from three independent experiments are depicted. Bars represent mean ± standard deviation from three independent experiments (>50 cells per experiment). The statistical significance (p value) was determined by a two tailed, paired Student's t-test. *$P < 0.05$. **(C)** Immunoblot of autophagy-related proteins in wildtype (ATG5^{WT}) and knockout (ATG5$^{-/-}$) immortalized baby mouse kidney epithelial cells (iBMKs) starved of glutamine for 24 h with or without BafA1. Numbers below the p62 immunoblot indicate the results of densitometric scanning of the protein bands. **(D)** Microscopic images of U2OS mCherry-ULK1 cells starved of glutamine for 24 h with or without WYE-125132 or BafA1. Scale bar = 10 μm. **(E)** Microscopic images of U2OS mCherry-ATG5/GFP-LC3 cells starved of glutamine for 24 h with or without WYE-125132 or BafA1. Error bars in all figures represent standard deviation of three biological replicates. Scale bar = 10 μm.

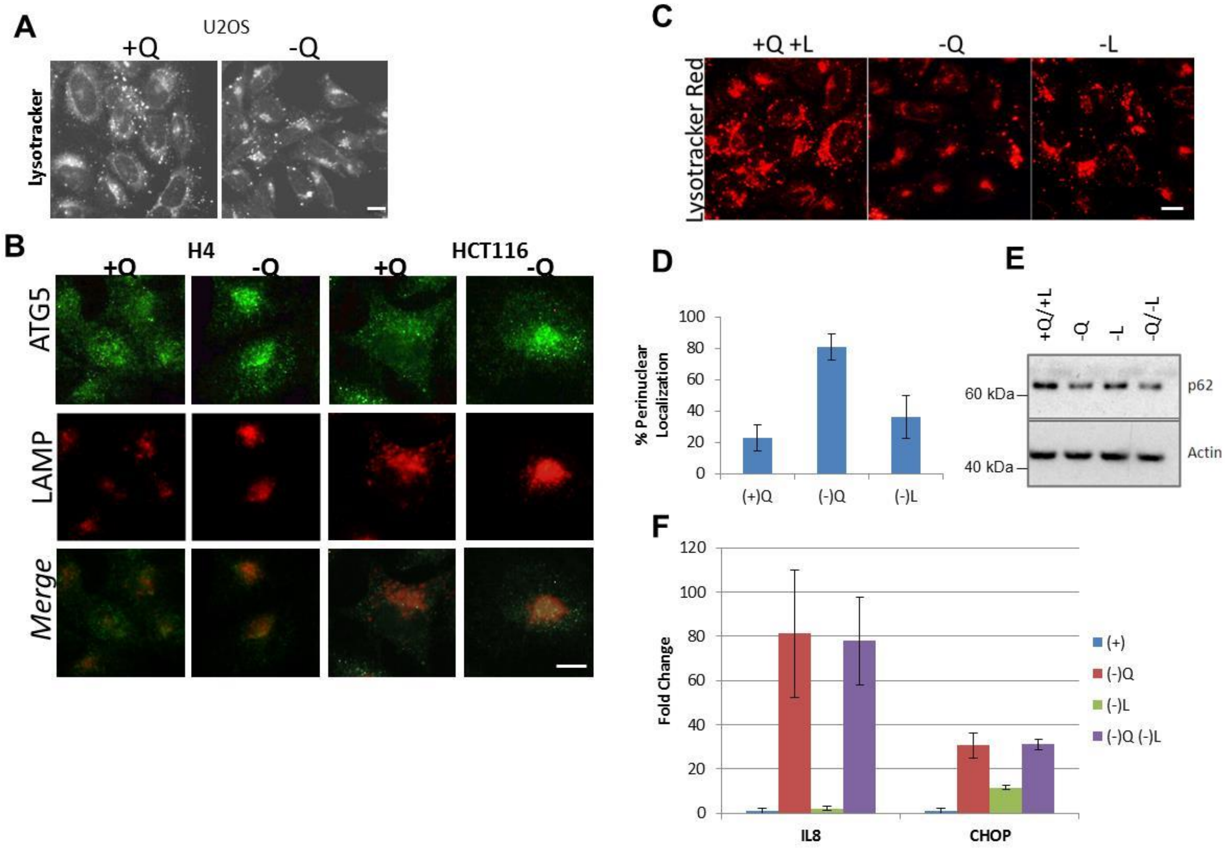

Supplementary Figure 3. Glutamine deprivation induces lysosome/autophagosome clustering.

(A) Lysotracker staining in U2OS cells cultured in media with or without glutamine (Q) for 24 h. Scale bar = 10 μm. **(B)** ATG5 and Lamp1 immunofluorescence in H4 neuroblastoma and HCT116 cells cultured with or without glutamine (Q) for 24 h. Scale bar = 10 μm. **(C)** Lysotracker staining in U2OS cells cultured in medium with or without glutamine (Q) or leucine (L) for 24 h. Scale bar = 10 μm. **(D)** Quantitation of images in (C). Error bars represent standard deviation. **(E)** Immunoblot of p62 in cells cultured in medium with or without glutamine (Q) or leucine (L) for 24 h. **(F)** RTPCR analysis of IL8 mRNA following 24 h glutamine (Q) or leucine (L) deprivation. Error bars in all figures represent standard deviation of three biological replicates.

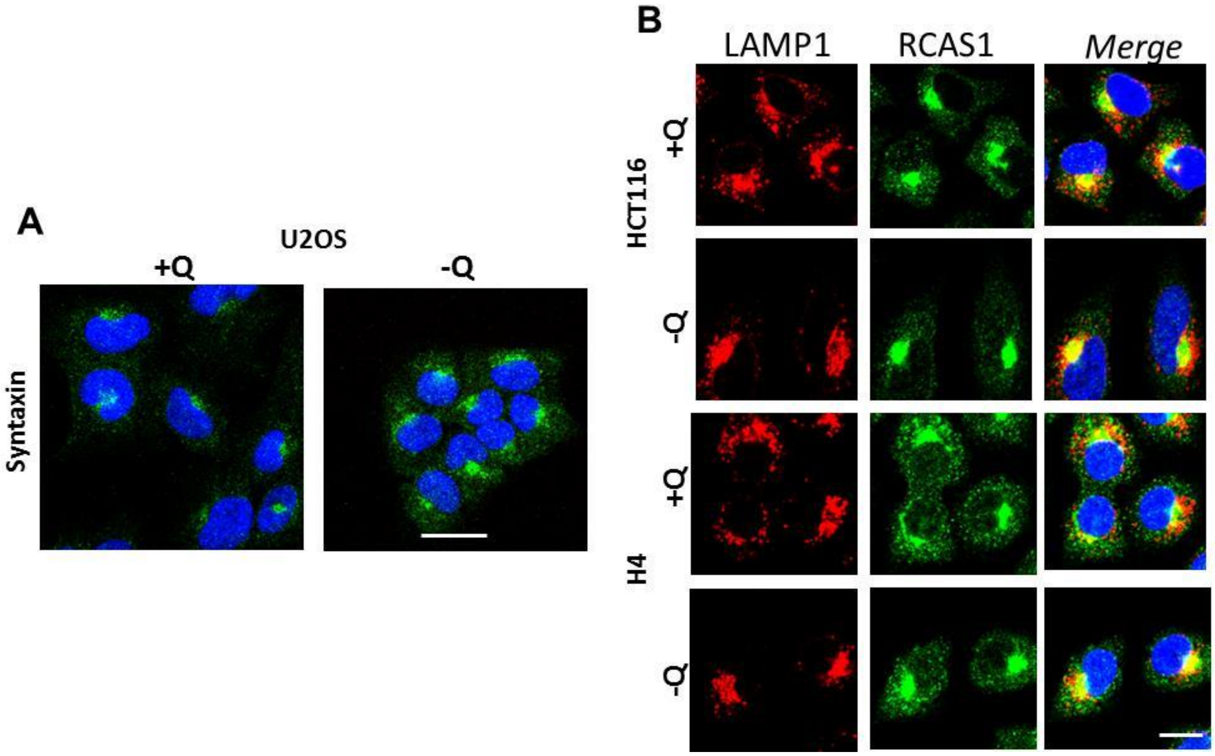

Supplementary Figure 4. Glutamine deprivation induces co-localization of Golgi membranes and lysosomes.

(A) Syntaxin immunofluorescence of U2OS cells starved of glutamine (Q) for 24 h. Scale bar = 30 μm. **(B)** Lamp1 and RCAS1 colocalization by immunofluorescence of glutamine-starved HCT116 and H4 cells. Scale bar = 15 μm.

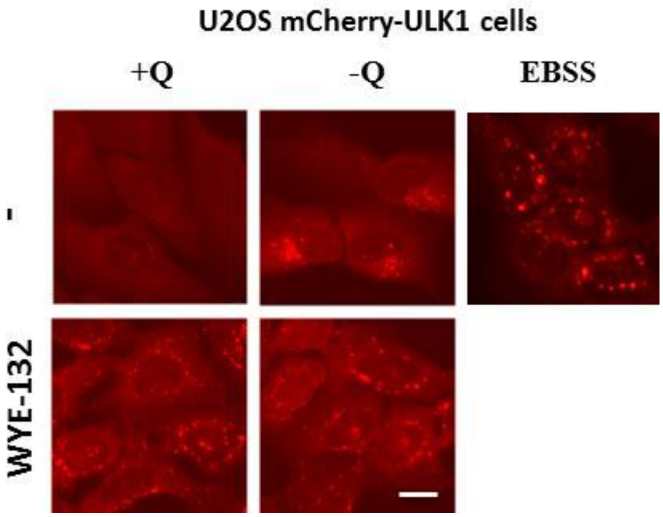

Supplementary Figure 5. Glutamine deprivation induces phagophore clustering that is dependent on mTOR activity.

U2OS-mcherry-ULK1 cells starved of glutamine or all amino acids (EBSS) for 24 h with or without WYE-125132. Scale bar = 10 μm.

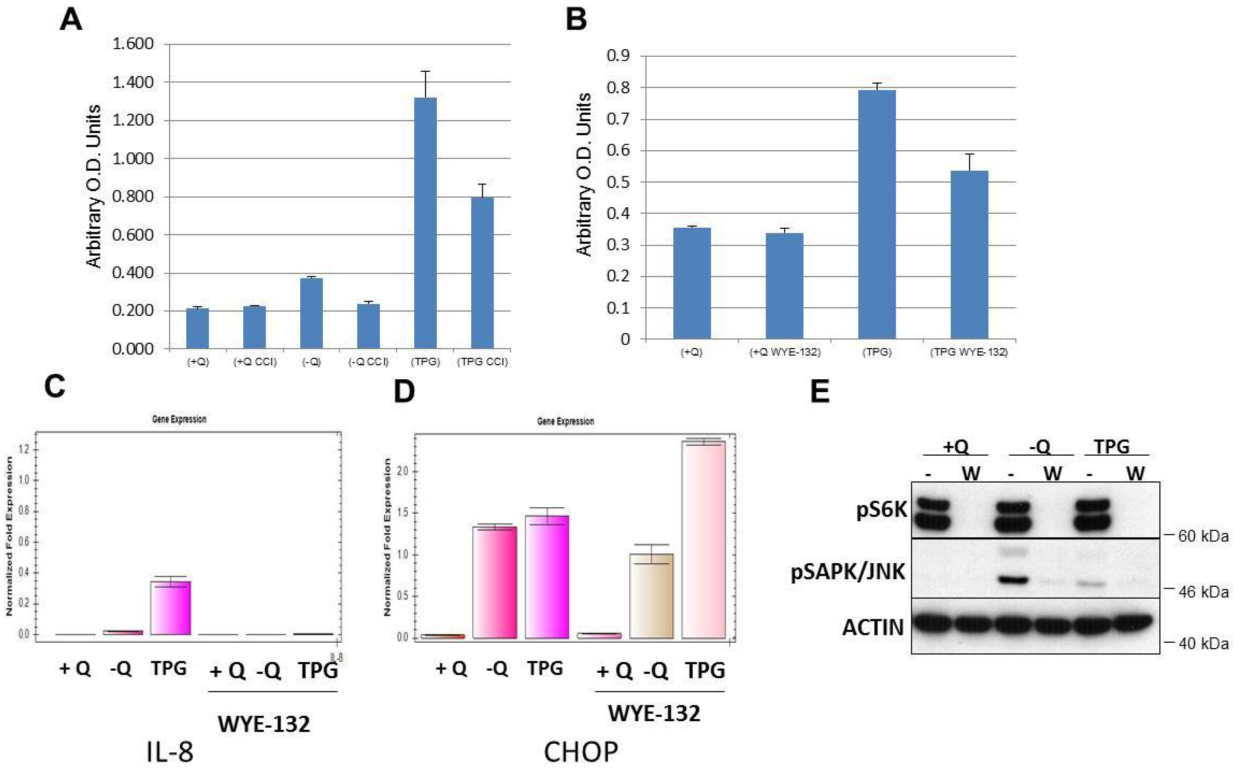

Supplementary Figure 6. Thapsigargin-induced ER stress partially mimics the responses to glutamine deprivation.

(A) IL-8 ELISA in U2OS cells starved of glutamine for 24 h or treated with 1 μM thapsigargin (TPG) with or without CCI-779. **(B)** IL-8 ELISA in U2OS cells treated with TPG with or without WYE-125132 for 24 h. **(C)** RT-PCR analysis of IL-8 mRNA expression in U2OS cells in the presence (+Q) or absence (-Q) of glutamine or TPG with or without WYE-132 after 24 h. **(D)** RT-PCR analysis of CHOP mRNA expression in U2OS cells in the presence (+Q) or absence (-Q) of glutamine or TPG with or without WYE-132 after 24 h. **(E)** Immunoblot analysis of pS6K and pSAPK/JNK in U2OS cells in the presence (+Q) or absence (-Q) of glutamine or TPG with or without WYE-125132 (W) for 24 h. Error bars in all figures represent standard deviation of three biological replicates.

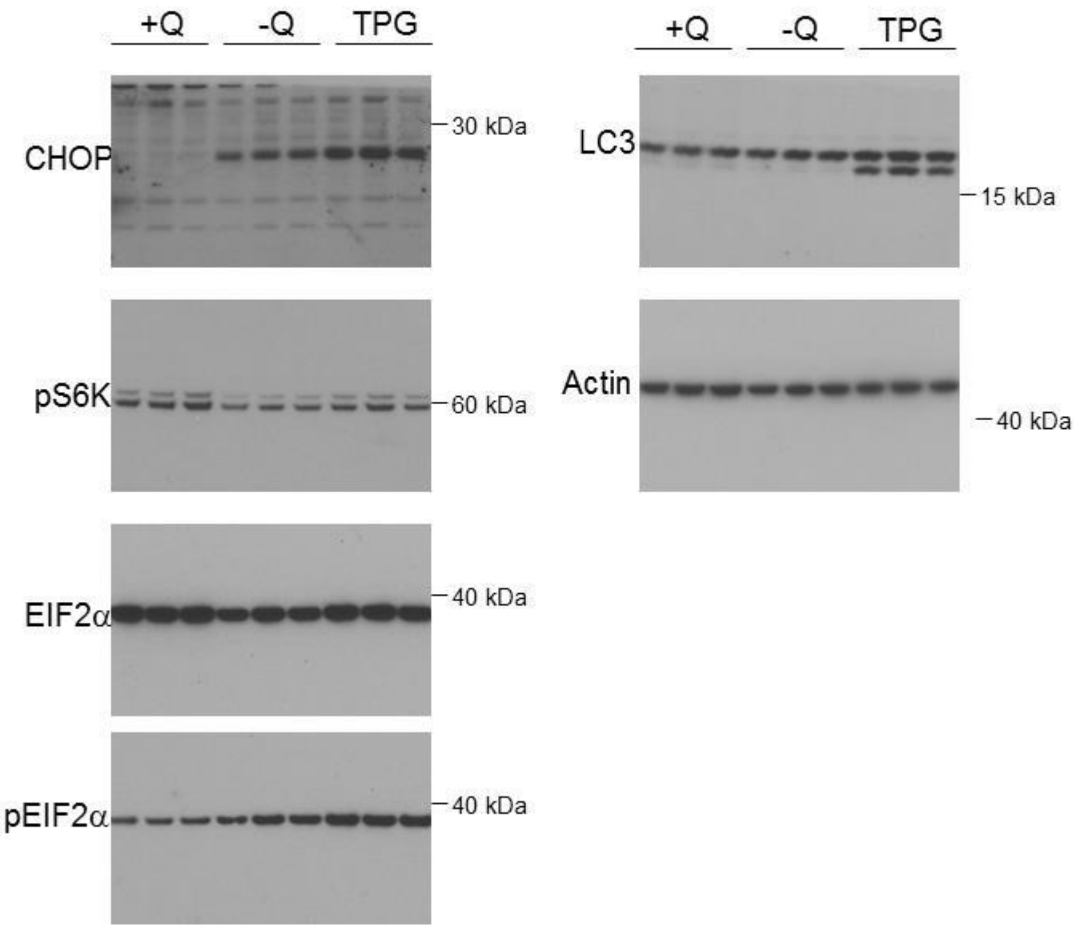

Supplementary Figure 7. Uncropped images of immunoblots shown in Figure 1B.

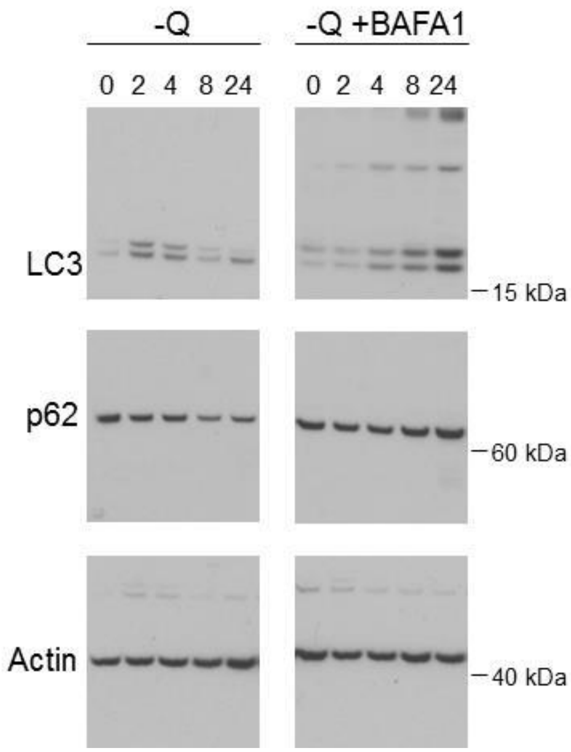

Supplementary Figure 8. Uncropped images of immunoblots shown in Figure 2C.

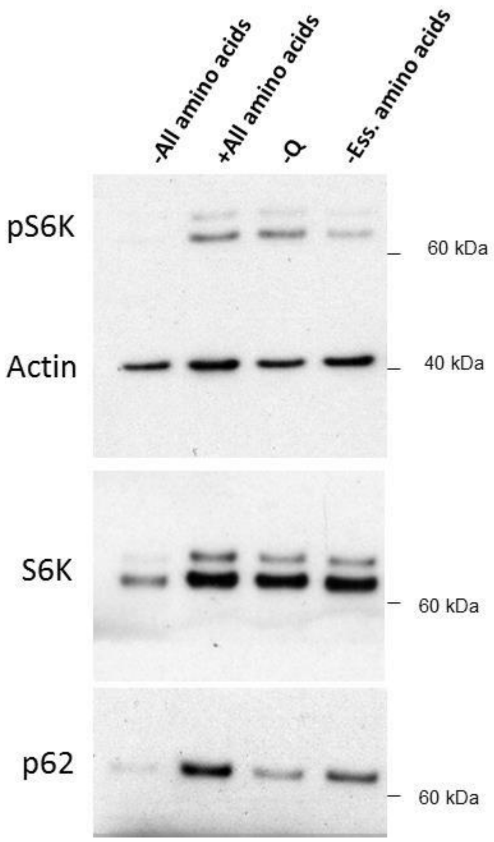

Supplementary Figure 9. Uncropped images of immunoblots shown in Figure 3E.

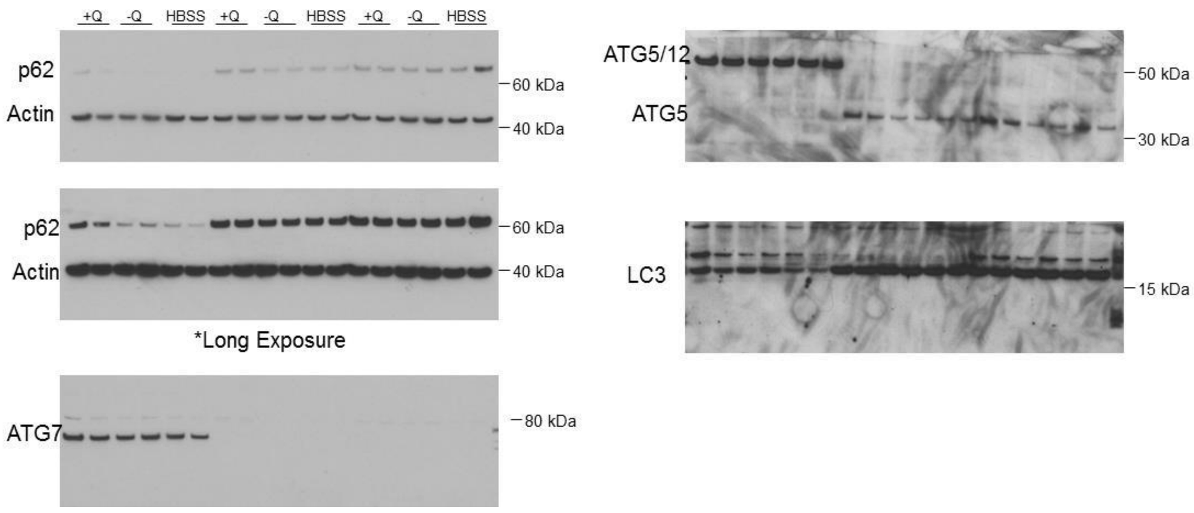

Supplementary Figure 10. Uncropped images of immunoblots shown in Figure 4A.

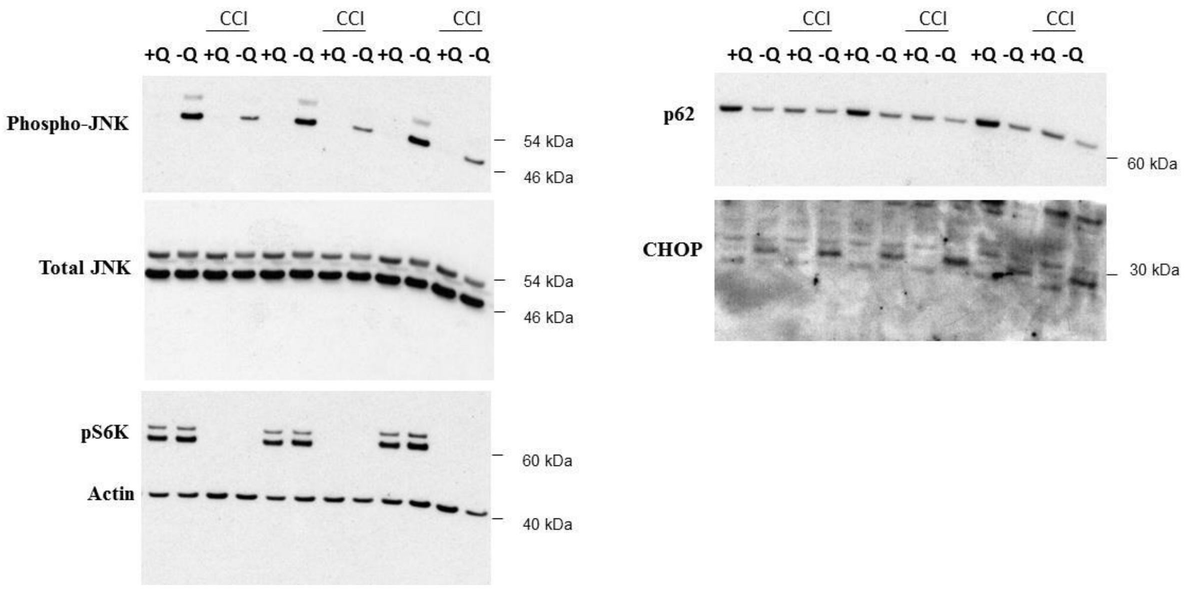

Supplementary Figure 11. Uncropped images of immunoblots shown in Figure 6A.

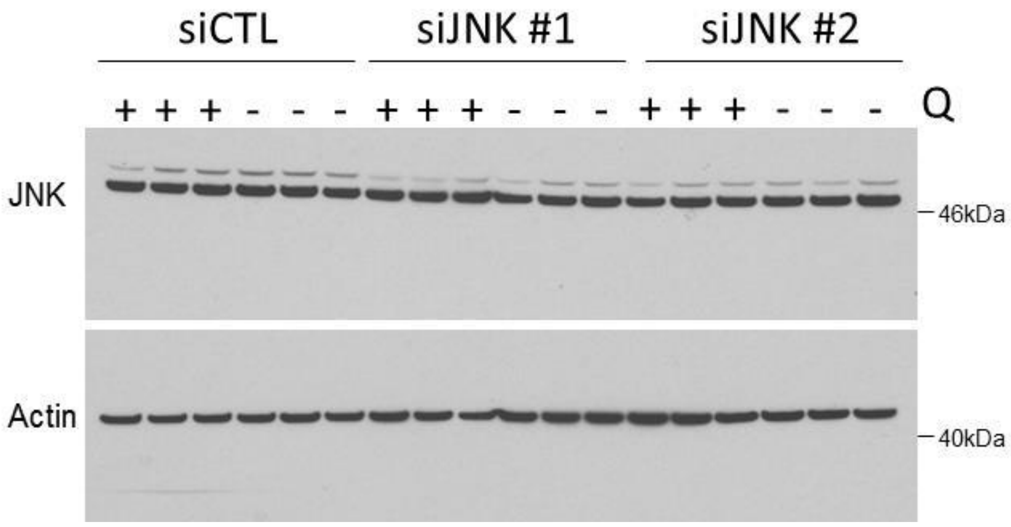

Supplementary Figure 12. Uncropped images of immunoblots shown in Figure 6D.

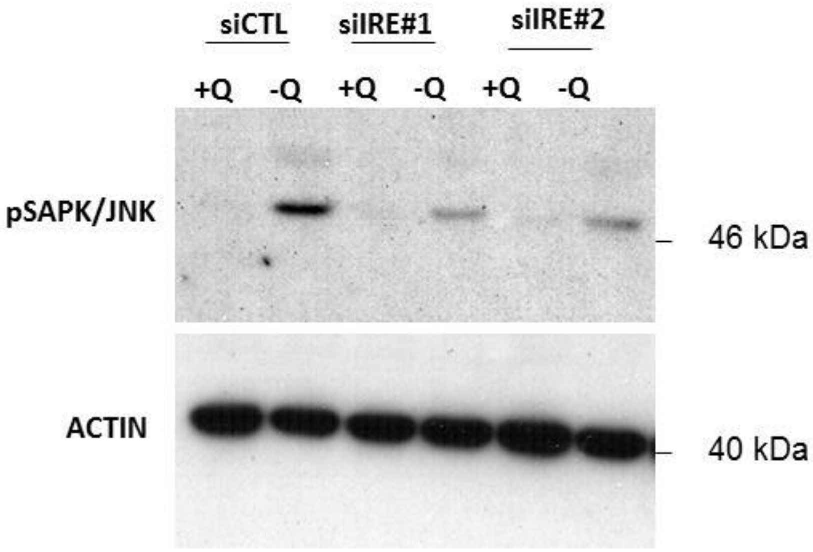

Supplementary Figure 13. Uncropped images of immunoblots shown in Figure 6K.

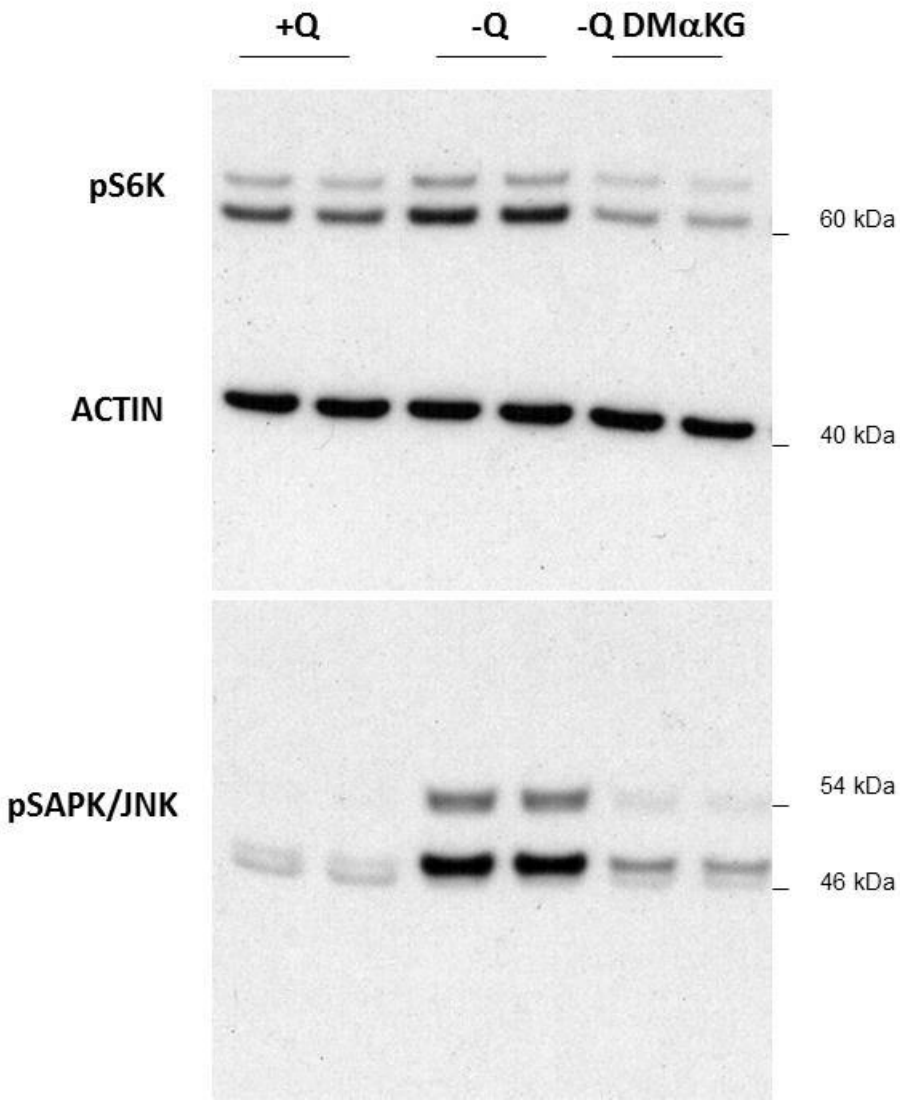

Supplementary Figure 14. Uncropped images of immunoblots shown in Figure 7E.
